# Proanthocyanidins protects 3-NPA-induced ovarian function decline by activating SESTRIN2-NRF2-mediated oxidative stress in mice

**DOI:** 10.1038/s41598-024-76743-w

**Published:** 2024-10-27

**Authors:** Yupei Huang, Yanfan Cui, Jian Huang, Huang Xinyuan, Wang Zihang, Tao Luo, Jia Li

**Affiliations:** 1 School of Basic Medical Sciences, Jiangxi Medical College, Nanchang University, Nanchang, 330031 Jiangxi China; 2 Institute of Biomedical Innovation, Nanchang University, Nanchang, 330031 Jiangxi China; 3 Clinical Medicine Center, Nanchang University, Nanchang, 330031 Jiangxi China

**Keywords:** Follicle atresia, SESTRIN2-NRF2 pathway, Oxidative stress, Ovarian function, Premature ovarian failure, Biochemistry, Cell biology, Drug discovery

## Abstract

Abnormal apoptosis of ovarian cells caused by oxidative stress is an important cause of premature ovarian failure (POF). Previous studies revealed that proanthocyanidins (PCs) are powerful natural antioxidants that can safely prevent oxidative damage in humans. However, the protective effect and mechanism of PCs on ovarian function during the course of POF remain unknown. In this study, female mice were injected with 3-nitropropionic acid (3-NPA) to establish an ovarian oxidative stress model; at the same time, the mice were treated with PC via gavage. Thereafter, the expression of various apoptosis genes, hormones, and related molecules was assessed. Compared with those in the control group, the ovarian index, follicle count at all levels, expression of MVH, PCNA and BCL2, and estradiol (E2) and progesterone (P) levels were significantly lower in the POF group, but significant recovery was observed in terms of MVH and PCNA expression and E2 and P levels in the POF + PCs group. The apoptosis marker genes BAX and ROS were significantly increased in the POF group but were notably restored in the POF + PCs group. In addition, the expression of Sestrin2, an antiapoptotic protein, was significantly increased in the PCs treatment group, as were the upstream and downstream regulatory factors NRF2 and SOD2, and the indices of the Sestrin2 overexpression group were similar to those of the PCs treatment group. In summary, these findings suggest that PCs have potential as innovative therapeutic agents for preventing and treating POF by activating the protective SESTRIN2-NRF2 pathway against oxidative stress.

**Supplementary Information:**

The online version contains supplementary material available at 10.1038/s41598-024-76743-w.

## Introduction

The classic definition of premature ovarian failure (POF) is the absence of menstruation for 4–6 consecutive months in women under 40 years of age, accompanied by elevated follicle-stimulating hormone (FSH) and decreased estradiol and anti-Müllerian hormone (AMH) levels^1^, and its incidence varies in different regions, with a number of studies reporting an incidence between 1%^2^ and 5.5%^3^, which is not only the main cause of ovarian infertility^4^ but also the clinical symptoms, such as endocrine disorders and perimenopausal syndrome, caused by this condition seriously affect women’s health^5^. Notably, abnormal apoptosis of ovarian granulosa cells (GCs) plays an important role in the pathogenesis and progression of POF^6^, whereas excessive accumulation of reactive oxygen species (ROS) resulting from oxidative stress emerges as a crucial factor contributing to granulosa cell death^7^. The most accepted model of ovarian aging also highlights the decreased ability of ovarian GCs to antagonize ROS^8^. Therefore, an increasing number of studies have investigated the involvement of oxidative stress (OS) in the onset and development of POF^9^.

Oxidative stress (OS) is caused by impaired cellular antioxidant defense mechanisms and excessive accumulation of intracellular ROS^10^. The primary sources of intracellular ROS are inflammation and mitochondria^11^. Normal levels of ROS play crucial roles in regulating follicular growth, angiogenesis and sex hormone synthesis in ovarian tissue^12^. However, when cellular antioxidant defense mechanisms are compromised, the newly produced ROS cannot be properly cleared, and excessive ROS disrupt mitochondrial integrity, leading to mitochondrial dysfunction and exacerbating ROS accumulation^13^. OS can directly or indirectly lead to POF in a variety of ways. OS can directly damage ovarian cells and reduce ovarian function^14^. Sha C et al.^15^ and Srinivas et al.^16^ reported that oxidative stress can directly induce cell DNA damage, interfere with the DNA damage response, reduce the acquisition of nutrients by ovarian cells, cause the apoptosis of GCs and oocytes, and lead to irreversible POF; OS can also damage GCs and oocytes by affecting mitochondrial function, leading to POF. Miao C et al.^17,18^have shown that the occurrence and development of POF are closely related to mitochondrial dysfunction, and when mitochondrial dysfunction leads to excessive ROS accumulation, mitochondrial genomic DNA lacks histone protection, antioxidant defense and an effective DNA repair system and is more vulnerable to damage^19^. OS can also lead to POF by affecting oocyte meiosis. Han L et al.^20^ reported that excessive ROS cause spindle defects, polar body abnormalities, and chromosomal mislocalization by acting on microtubules to attach to centroenoids. In addition, oxidative stress can cause DNA double-strand breaks, hinder oocyte meiosis progression to metaphase II (MII), and inhibit follicle maturation^21,22^. ROS play a direct role in this process, so it is possible to reduce and treat POF by eliminating ROS with antioxidants. Several studies have focused on investigating the effects of antioxidants on POF^23,24^.

Proanthocyanidins (PCs), which are effective natural antioxidants that can be obtained from the seeds of many common plants^25^, are widely used because of their antioxidant, anti-inflammatory, antiangiogenic, antiproliferation and immunomodulatory effects^26,27^. For example, research has demonstrated that PCs can protect against liver injury in mice caused by oxidative stress by activating the NRF2/ARE signaling pathway^28^. In addition, proanthocyanidin B2 can effectively mitigate the oxidative stress induced by oocyte vitrification, restore mitochondrial function, and improve oocyte viability. Moreover, it acts as a corticotonic regulator, maintains normal spindle morphology, promotes migration to ensure the correct meiosis process, and ultimately reduces the aneuploidy rate of vitrified oocytes^29^.

Sestrins (SESNs), a highly conserved protein family consisting of three members (Sestrin1–3) in vertebrates^30^, respond to a variety of environmental stresses, including DNA damage-related oxidative and nutritional stresses^31^. Sestrins affect a variety of signaling pathways, such as the mTORC and REDOX signaling pathways, which play important roles in aging^32^. There is evidence that Sestrins can reduce ROS^33^ and inhibit mTORC^34^ activity, suggesting that this protein family may exert an antiaging effect by inhibiting these two well-characterized aging promoters^31^. In our previous studies, we reported that the expression of the Sestrin family is high in ovarian tissue and that sestrin2 is closely related to oxidative stress^35^; however, the mechanism underlying the action of Sestrin2 remains unclear.

Nuclear factor-E2-associated factor-2 (NRF2) is an emerging regulator of the cellular antioxidant response that governs the expression of a cascade of antioxidant genes^36^. NRF2 is regulated primarily by Keap1^37^, and oxidative damage triggers Keap1 degradation and subsequent NRF2 activation^38^. NRF2 is translocated into the nucleus to orchestrate the cell’s defense against oxidative damage. There is evidence supporting NRF2’s ability to specifically upregulate SESTRIN2 transcription levels^39,40^.

Therefore, the purpose of this study was to investigate the potential of the protective SESTRIN2-NRF2 pathway by PCs in the prevention and treatment of POF by activating against oxidative stress.

## Materials and methods

### Ethical approval

All mice (the Kunming strain) were provided by the Department of Animal Science of Nanchang University, and the experiments were conducted in accordance with the National Institutes of Health Guide for the Care and Use of Laboratory Animals. This study was approved by the Animal Ethics Committee of Nanchang University (NCU2022101401). All mice were anesthetized with carbon dioxide and euthanized by carbon dioxide overdose inhalation.

### Model establishment

The animals were raised in a conventional facility (12 h light/dark cycle, 23 ± 1 °C, 45%±5% relative humidity, and free access to distilled water and commercial rodent food ad libitum) for 1 week before the experiment. The mice were randomly divided into four groups of four groups (*n* = 5): the control group (NaCl group), PCs group, POF group, and treatment group (PCs + POF group). The POF group received continuous intraperitoneal injections of 3-NPA (12.5 mg/kg/day, 164603, Sigma‒Aldrich) for 14 days^41^; the POF + PCs group was injected intraperitoneally with 3-NPA for 14 days and continuously intragastrically administered PCs (200 mg/kg/day, 7230, Solarbio) for 14 days; the control group and PCs group were intragastrically administered NaCl (0.9%) and PCs, respectively, for 14 days.

### Ovarian index and hormone determination

The body weights of the mice were recorded. The ovaries were cleaned with a phosphate-buffered saline (PBS) solution. The ovarian surface fluid was removed with absorbent paper, and the samples were weighed. The ovarian index was calculated via the following formula: ovarian index (‰) = ovarian weight/body weight * 1000.

Blood was collected from the eyeballs for hormone determination. The levels of FSH (E-EL-M0511c, Elabscience, China), estradiol (E2) (E-EL-0150c, Elabscience, China), progesterone (P) (E-OSEL-M0006) and anti-Müllerian hormone (AMH) (E-EL-M3015, Elabscience, China), SOD (E-EL-H6188, Elabscience, China), GSH (E-EL-0026, Elabscience, China), 8-OHdG (E-EL-0028, Elabscience, China), and 4-HNE (E-EL-0128, Elabscience, China) were assessed by corresponding enzyme-linked immunosorbent assays (ELISAs) according to the manufacturers’ instructions.

### Histological analysis of ovarian tissue and ovarian follicle counts

Mouse ovaries were fixed with 4% paraformaldehyde solution, dehydrated in ethanol and xylene, and embedded in paraffin. The paraffin-embedded ovaries were serially sectioned at a thickness of 5 μm with a microtome (RM2255, Leica, Berlin, Germany). The ovary sections were then dewaxed in xylene, rehydrated in ethanol, and stained with H&E^42^. The ovarian follicles were counted as previously described^43^. All the sections were viewed with an Olympus IX73 microscope.

### Immunohistochemistry (IHC)

IHC was carried out on 5 μm sections of paraffin-embedded tissue. After the slides were baked, dewaxed, and rehydrated, antigen retrieval was performed. The primary antibodies used for IHC were anti-BCL2 (1:1000,26593-1-AP), anti-BAX (1:5000,50599-2-Ig), anti-KEAP1 (1:500,10503-2-AP), anti-MVH (1:1000,51042-1-AP), anti-SESTRIN2 (1:500,10795-1-AP), anti-NRF2 (1:500,16396-1-AP), anti-SOD2 (1:2000,24127-1-AP), and anti-PCNA (1:4000,10205-2-AP) antibodies. The secondary antibody and visualized stain for peroxidase visualization were performed via a DAB substrate kit (PV-6000D, ZSGBbio, Beijing, China), and the slides were counterstained with hematoxylin (AR1180-100, Boster). To confirm the validity and reliability of the reagents used for IHC staining, especially specific antibodies, negative controls were used.

### Western blot

Total protein was extracted via the TRIzol method. Volumes of supernatant containing equivalent amounts of total protein (5–10 µL) were subjected to SDS‒PAGE. The MVH, SESTRIN2, NRF2, KEAP1, SOD2, BCL2, BAX and PCNA proteins were visualized via rabbit MVH (1:750), rabbit SESTRIN2 (1:1000), rabbit NRF2 (1:500), mouse KEAP1 (1:2000), rabbit SOD2 (1:2000), rabbit BCL2 (1:1000), rabbit BAX (1:5000) and rabbit PCNA (1:2000) antibodies, as well as the anti-rabbit-HRP antibody or anti-mouse-HRP antibody at a 1:5000 dilution. The relative levels of the target proteins were analyzed via ImageJ software (version 1.44; National Institutes of Health, Bethesda, MD, USA). Each experiment was repeated at least three times.

### Reactive oxygen species (ROS) determination

The ovaries were crushed, and PBS was added. The mixture was collected, and the supernatant was removed and washed with PBS twice. DCHF-DA mixture was added, and the mixture was incubated at 37 °C for 30 min. The probes were then fully connected with the cells. The suspension was collected and centrifuged again and then placed into an enzyme-labeled analyzer at a wavelength of 480 nm^44^.

### Reverse transcription‒quantitative PCR

RNA was extracted via the TRIzol method. The expression levels of *Mvh*, *Pcna*, *Sestrin2*, and *Nrf2* in each sample were measured via real-time quantitative reverse transcription PCR (qRT‐PCR) with PCR Master Mix (novoprotein E096-01, E047-01) according to the manufacturer’s instructions and standardized to the relative expression levels of the target gene/β‐actin. The cycling conditions were as follows: 95 °C for 2 min, 35 cycles of denaturation at 95 °C for 15 s, annealing at 55 °C for 30 s, and extension at 68 °C for 1 min. All reactions were repeated three times. The resulting amplification and melting curves were analyzed to ensure the identity of the specific PCR products. Threshold cycle values were used to calculate the fold change in the transcript levels via the 2ΔΔCt method.

### Microinjection of lentivirus in vivo

All the mice were anesthetized with 1% sodium pentobarbital via intraperitoneal injection and then fixed in the prone position. A 1.0 cm longitudinal incision was cut at the side of the spine through the subcutaneous tissue, muscular layer, and peritoneum. Both ovaries were carefully removed under a microscope, tweezers were used to pull up the ovarian membranes, and SESTRIN2-overexpressing (SESN2-OE) lentivirus (3.5 × 10^8^ TU/mL) was injected into the ovarian capsule of POF under a microscope^45^. In the simulation group, the lentivirus was replaced with an equal amount of empty lentiviral vector. The ovaries were placed back into the abdomen after injection, and the incision was sterilized and sutured. The mice in each group were allowed to acclimate to the environment for 1 week.

### Definition of the phase of the estrous cycle

To determine the phase of the estrous cycle, vaginal smears of the mice in the three groups were taken at approximately 9 am every day for 11 consecutive days. The stage of the estrous cycle is judged by cytology under a microscope, and the estrous cycle is divided into proestrus, oestrus, and metestrus. Smears were taken continuously for 11 days^46^. Moreover, the ovaries were stained with hematoxylin and eosin (H&E), macroscopic and microscopic analyses were performed, and the ovaries were categorized as being in the follicular phase.

### Statistical analysis

The experimental results are expressed as the mean ± SEM and were statistically analyzed with GraphPad Prism 8 software (GraphPad Software, Inc., San Diego, CA, USA). The analysis methods used were an unpaired t test and one-way ANOVA with Tukey’s test. *P* < 0.05, *P* < 0.01 and *P* < 0.001 were considered to indicate statistical significance. Each experiment was repeated at least three times.

## Results

### Proanthocyanidins protect against 3-NPA-induced ovarian follicular development impairment

**Fig. 1:**
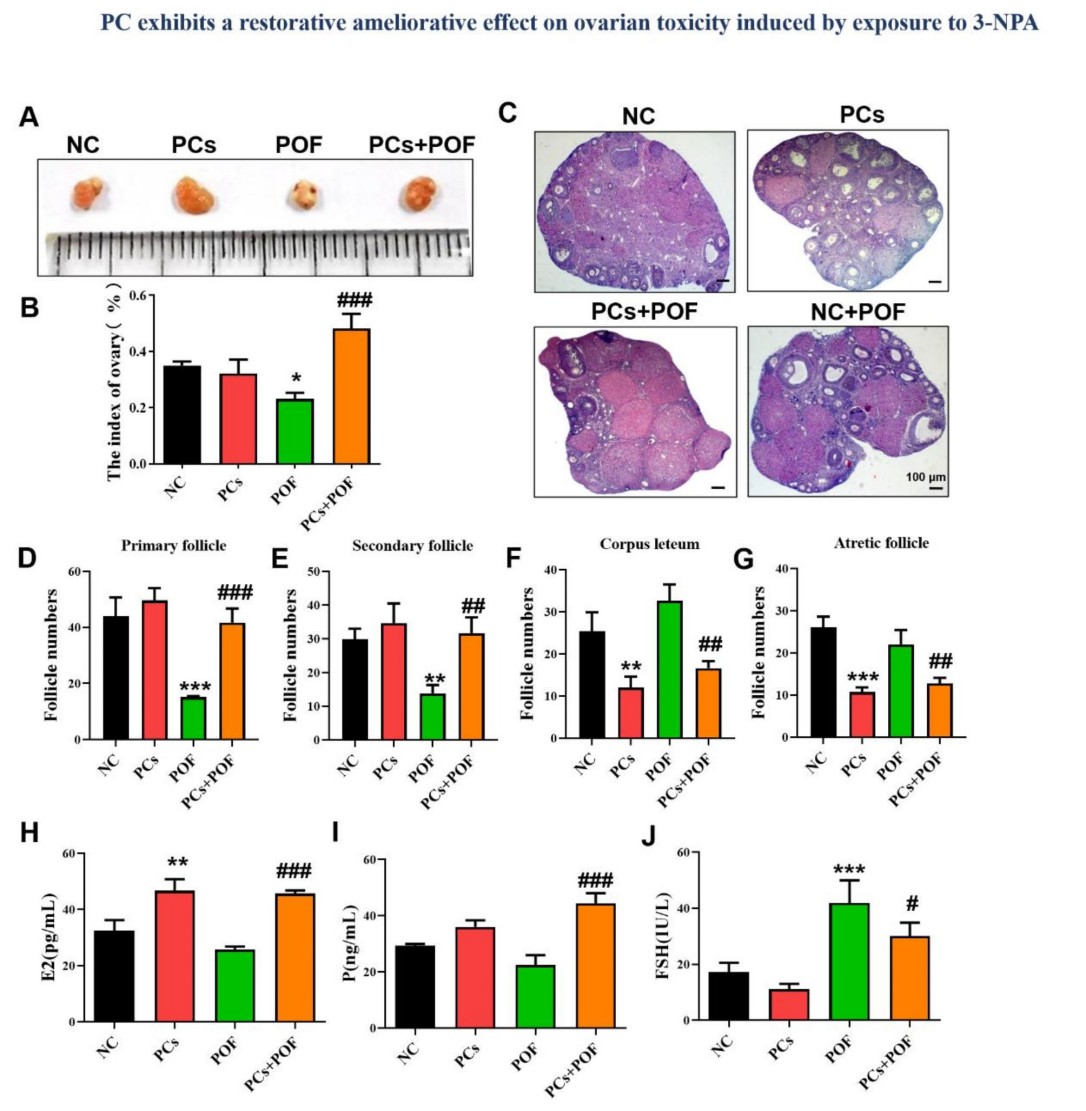
Effects of PCs on ovarian weight, microstructure and follicle development. (**A**) Differences in ovarian appearance among the NC, PCs, POF and PCs + POF groups. (**B**) Effects of PCs on the ovary index (ovary weight/body weight, mg/g). (**C**) The morphologies of ovaries in the NC, PCs, POF and PCs + POF female mice were examined after hematoxylin‒eosin (HE) staining. (**D**-**G**) Statistical analyses of primary follicles, secondary follicles, corpus lutea and atretic follicles in the NC (*n* = 3), PCs (*n* = 3), POF (*n* = 3) and PCs + POF (*n* = 3) groups were performed according to the HE staining results. (**H**-**J**) Hormone levels of E2, P and FSH in the peripheral blood of the mice. *P < 0.05, **P < 0.01, and ***P < 0.001 vs. the NC group; #*P* < 0.01, ##*P* < 0.01 and ###*P* < 0.001 vs. the POF group; one-way ANOVA.

The 3-NPA-induced ovarian function damage model was established to determine the effect of PCs on ovarian injury. As shown in Fig. 1A, B, the ovarian weight and index increased in the POF + PCs group compared with those in the POF group. H&E staining was used to observe the changes in ovarian structure in each group (Fig. 1C). Compared with those in the NC group, the corpus luteum and atretic follicles were significantly reduced in the PCs group, the structure was destroyed, and the number of primary and secondary follicles was significantly reduced in the POF group (Fig. 1. C-G; *P* < 0.01). However, HE staining revealed that after PCs treatment, the number of primary and secondary follicles increased significantly, whereas that of the corpus luteum and atretic follicles decreased (Fig. 1. C-G; *P* < 0.01). In addition, compared with those in the NC group, the content of E2 in the PCs group was significantly increased, while the contents of P and FSH were not significantly different (Fig. 1. H-J; *P* < 0.01). The E2 and P levels in the POF group did not significantly differ, whereas the FSH content significantly increased, but the levels recovered after PCs treatment (POF + PCs group) (Fig. 1. H-J; *P* < 0.05).

### Proanthocyanidins restore excessive 3-NPA-induced ovarian apoptosis and promote reproductive cell proliferation

**Fig. 2:**
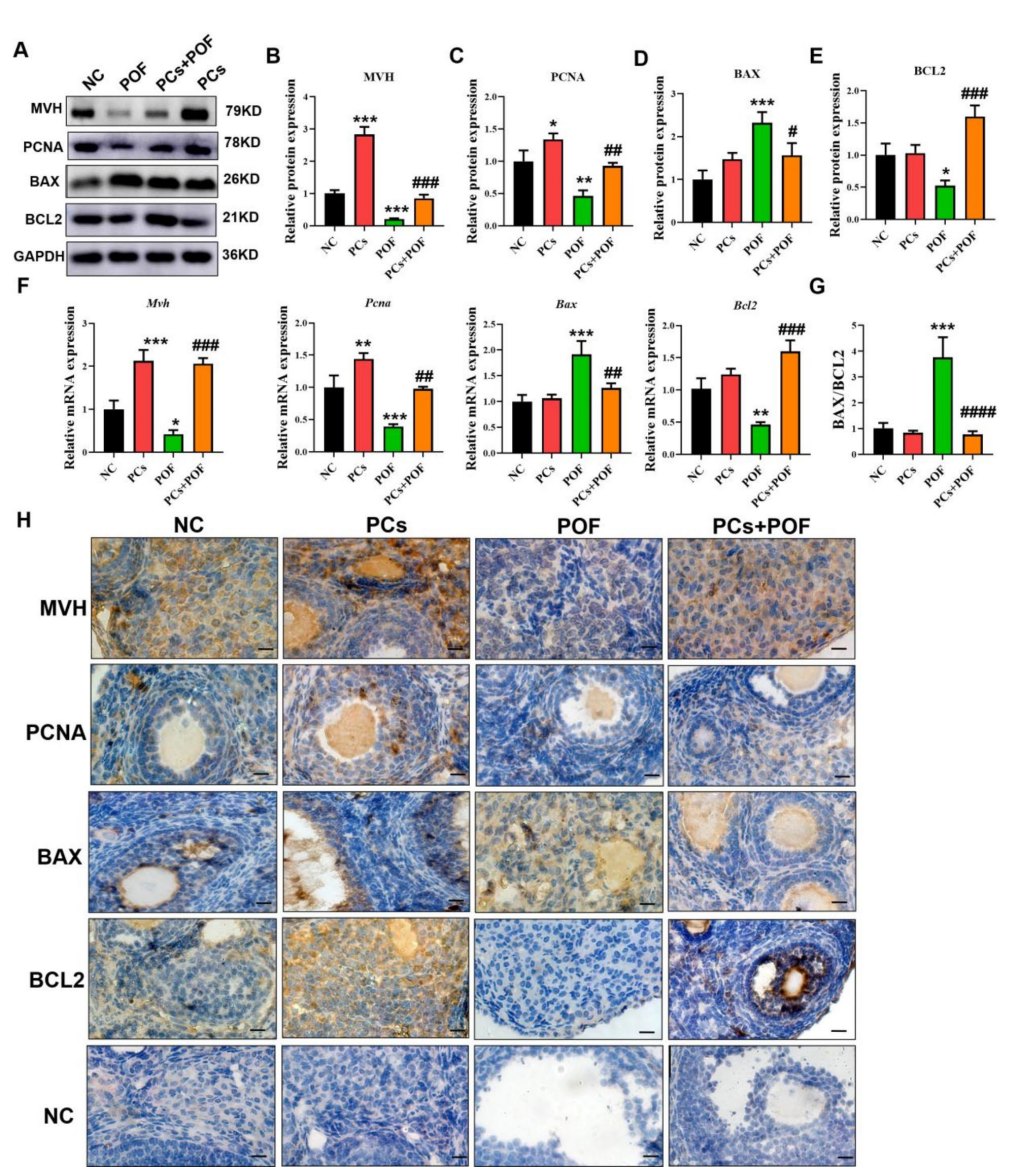
Effects of PCs on ovarian cell apoptosis and impaired gene expression. (**A**-**E**) Western blot analysis of MVH-, PCNA-, BAX- and BCL2-related proteins in the NC, PCs, POF and PCs + POF groups. (**F**) Expression levels of *Mvh*, *Pcna*, *Bax* and *Bcl2* mRNAs in the NC, PCs, POF and PCs + POF groups. (**G**) The *Bax*/*Bcl2* ratio. (**H**) IHC results of MVH, PCNA, BAX and BCL2 in the NC, PCs, POF and PCs + POF groups. The scale bar represents 50 μm. *P < 0.05, **P < 0.01 and ***P < 0.001 vs. the NC group; #*P* < 0.01, ##*P* < 0.01 and ###*P* < 0.001 vs. the POF group; one-way ANOVA.

The degree of apoptosis of the ovarian cells in each group was compared by measuring the protein expression levels of multiple apoptosis-related genes (Fig. 2. A-E; *P* < 0.05). Compared with those in the NC group, the protein expression levels of MVH and PCNA were significantly increased in the PCs group, while there was no significant change in the expression levels of BAX and BCL2. The protein expression of MVH, PCNA and BCL2 in the POF group was significantly decreased, and the protein expression of the BAX protein was significantly increased. Compared with those in the POF group, the protein expression levels of MVH, PCNA and BCL2 were significantly increased, and the protein expression level of BAX was significantly decreased in the POF + PCs group. Compared with those in the NC group, the mRNA expression levels of *Mvh* and *Pcna* were significantly increased in the PCs group, whereas the mRNA expression levels of *Bax* and *Bcl2* were not significantly different. The mRNA expression levels of *Mvh*, *Pcna* and *Bcl2* in the POF group were significantly decreased, whereas the mRNA expression level of *Bax* was significantly increased. Compared with those in the POF group, the mRNA expression levels of *Mvh*, *Pcna* and *Bcl2* in the POF + PCs group were significantly increased, while the mRNA expression levels of *Bax* and the *Bax*/*Bcl2* ratio were significantly decreased (Fig. 2. F, G; *P* < 0.01). In each group, the results of IHC were similar to those of western blot analysis of MVH, PCNA, BAX and BCL2-related proteins (Fig. 2H). These results indicate that PCs can protect ovaries by inhibiting cell apoptosis, promoting reproductive cell proliferation and regulating sex hormone secretion.

### Proanthocyanidins protect ovarian function by inhibiting oxidative stress through the SESTRIN2-NRF2 pathway

**Fig. 3:**
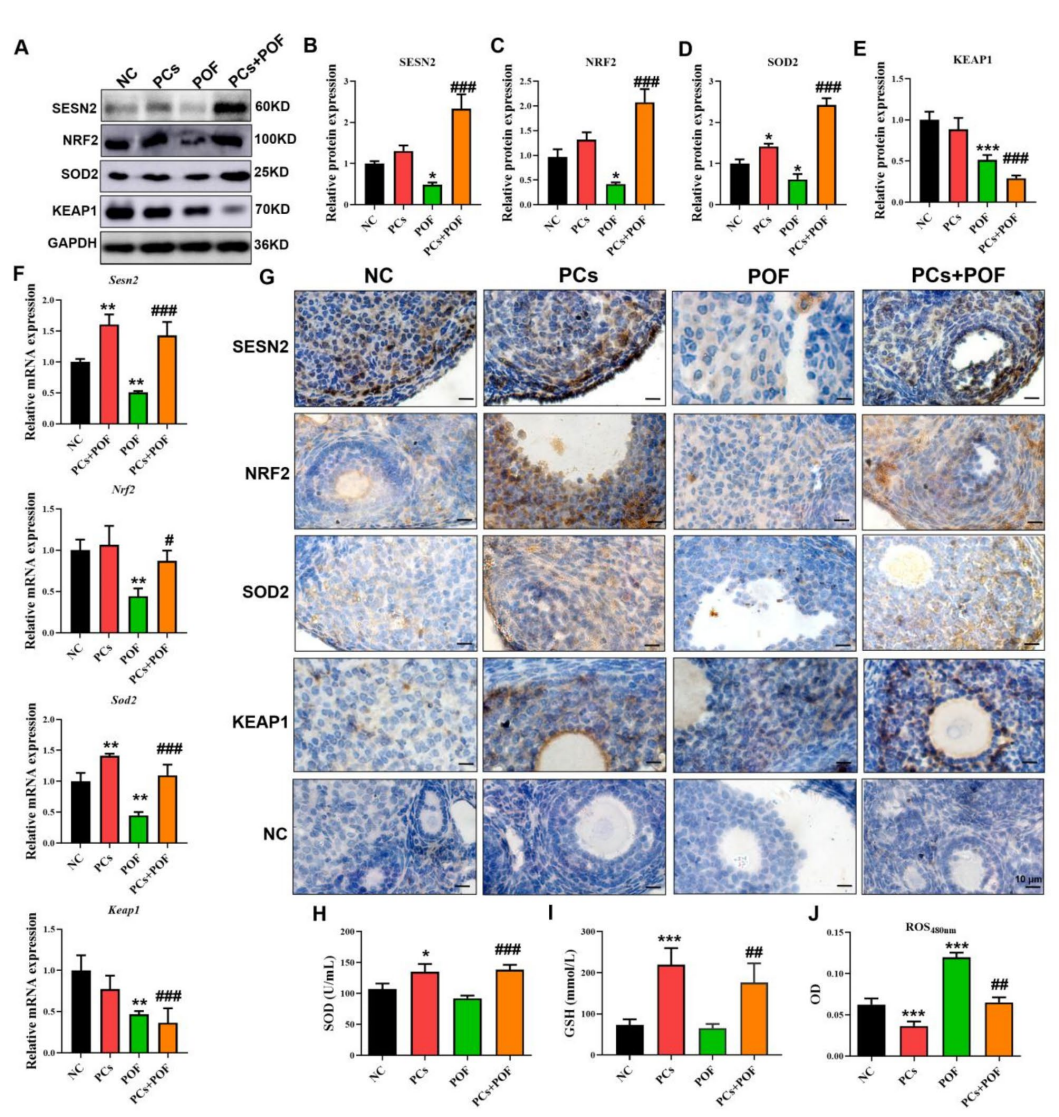
PCs improved the activity of the SESTRIN2-NRF2 signaling pathway and reduced oxidative stress in POF models. (**A**-**E**) Relative protein expression levels of SESTRIN2-, NRF2-, SOD2- and KEAP1-related proteins in the NC, PCs, POF and PCs + POF groups. (**F**) Expression levels of *Sestrin2*, *Nrf2*, *Sod2* and *Keap1* mRNAs in the NC, PCs, POF and PCs + POF groups. (**G**) IHC results of SESTRIN2, NRF2, and SOD2 in the NC, PCs, POF and PCs + POF groups. (**H**, **I**) Determination of the GSH and SOD hormone levels in the peripheral blood. (**J**) ROS were detected by a fluorescent DCFH-DA probe in a luciferase plate analyzer, and the OD values were recorded at 480 nm. The scale bar represents 50 μm. *P < 0.05, **P < 0.01 and ***P < 0.001 vs. the NC group; #*P* < 0.01, ##*P* < 0.01 and ###*P* < 0.001 vs. the POF group; one-way ANOVA.

SESTRIN2 signaling is a cellular energy and signal sensor that regulates many crucial cellular processes, including apoptosis^47^. Moreover, SESTRIN2 is involved in the regulation of ovarian function^48^. We therefore evaluated the role of the SESTRIN2 signaling pathway in the ability of PCs to protect ovarian function by inhibiting oxidative stress in mice. As shown in Fig. 3A-E, PCs treatment increased the expression of SESTRIN2-NRF2 and increased the expression of SOD in ovarian cells (*p* < 0.05). However, compared with those in the POF group, the levels of SESTRIN2, NRF2 and SOD2 in the POF + PCs group increased significantly, but the levels of KEAP1 tended to decrease. In addition, compared with those in the NC group, the mRNA expression levels of *Sestrin2* and *Sod2* in PCs group were significantly increased, while the mRNA expression levels of *Nrf2* and *Keap1* were not significantly different. The mRNA expressions levels of *Sestrin2*, *Nrf2*, *Sod2* and *Keap1* in the POF group were significantly lower, and the mRNA expression levels of *Sestrin2*, *Nrf2* and *Sod2* in the POF + PCs group were significantly increased compared with those in the POF group (Fig. 3. F; *P* < 0.01). In each group, the results of IHC were similar to those of western blot analysis of MVH-, PCNA-, BAX- and BCL2-related proteins (Fig. 3G). Importantly, compared with those in the NC group, the contents of SOD and GSH in the PCs group were significantly increased, and the absorbance of ROS was significantly lower; however, the contents of SOD and GSH in the POF group were not significantly different, and the absorbance of ROS was significantly increased. Compared with those in the POF group, the SOD and GSH contents in the POF + PCs group were significantly increased, and the ROS absorbance was significantly decreased (Fig. 3. H-J; *P* < 0.05). These results confirmed that PCs activated the SESTRIN2-NRF2 pathway in POF mice, alleviating oxidative stress.

### SESTRIN2 overexpression recovers reproductive endocrine function in POF mice

**Fig. 4:**
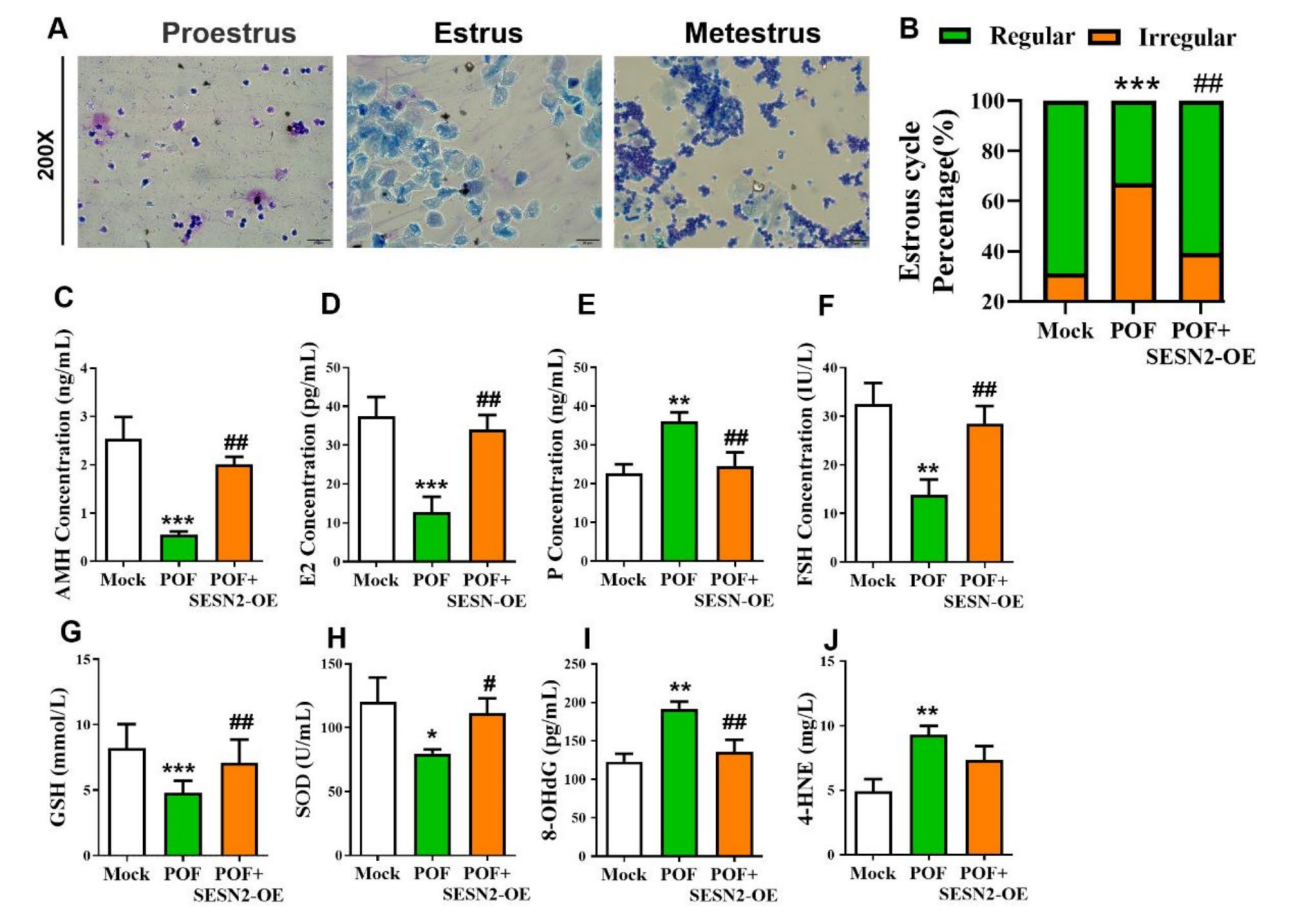
Effect of SESN2-OE on reproductive endocrine function in POF mice. (**A**) Analysis of the estrous cycle via smears of exudated cells from the mouse vagina. (**B**) Proportion of regular or irregular estrous cycles in the mice.  In the Mock, POF, and POF + SESN2-OE groups (*n* = 5). (**C**–**F**) The AMH (**C**), E2 (**D**), P (**E**) and FSH (**F**) levels of the mice in the Mock, POF, and POF + SESN2-OE groups (*n* = 5) were examined via enzyme-linked immunosorbent assay. (**G**–**J**) Determination of the GSH, SOD, 8-OHdG and 4-HNE hormone levels in the peripheral blood. **p  < 0.01, ***p  < 0.001, compared with the Mock group; ## *p* < 0.01, compared with the POF group; one-way ANOVA.

As shown in Fig. 4, mouse vaginal exfoliated cells were observed via the smear method to analyze the estrus cycle of the mice, and the proportions of regular estrus in the mock group, POF group and POF + SESN2-OE group were calculated. The results revealed that the irregular proportion increased significantly in the POF group, whereas the regularity rate recovered significantly in the overexpression treatment group (Fig. 4. A, B; *P* < 0.01). Comparisons of the levels of multiple sex hormones in the three groups of mice (Fig. 4. C-F; *P* < 0.01) showed that the levels of various sex hormones in the overexpression treatment group significantly returned to normal. These results indicated that the overexpression of SESTRIN2 recovered reproductive endocrine function in POF mice. A comparison of the levels of oxidative stress-related factors among the three groups of mice revealed that (Fig. 4. G‒J; *P* < 0.01) showed that SESTRIN2 overexpression group significantly increased the level of antioxidant enzymes and significantly decreased the degree of oxidative stress, suggesting that SESTRIN2 overexpression can inhibit ovarian oxidative stress in POF mice.

### Overexpression of SESTRIN2 promotes follicle development and ovarian function

**Fig. 5:**
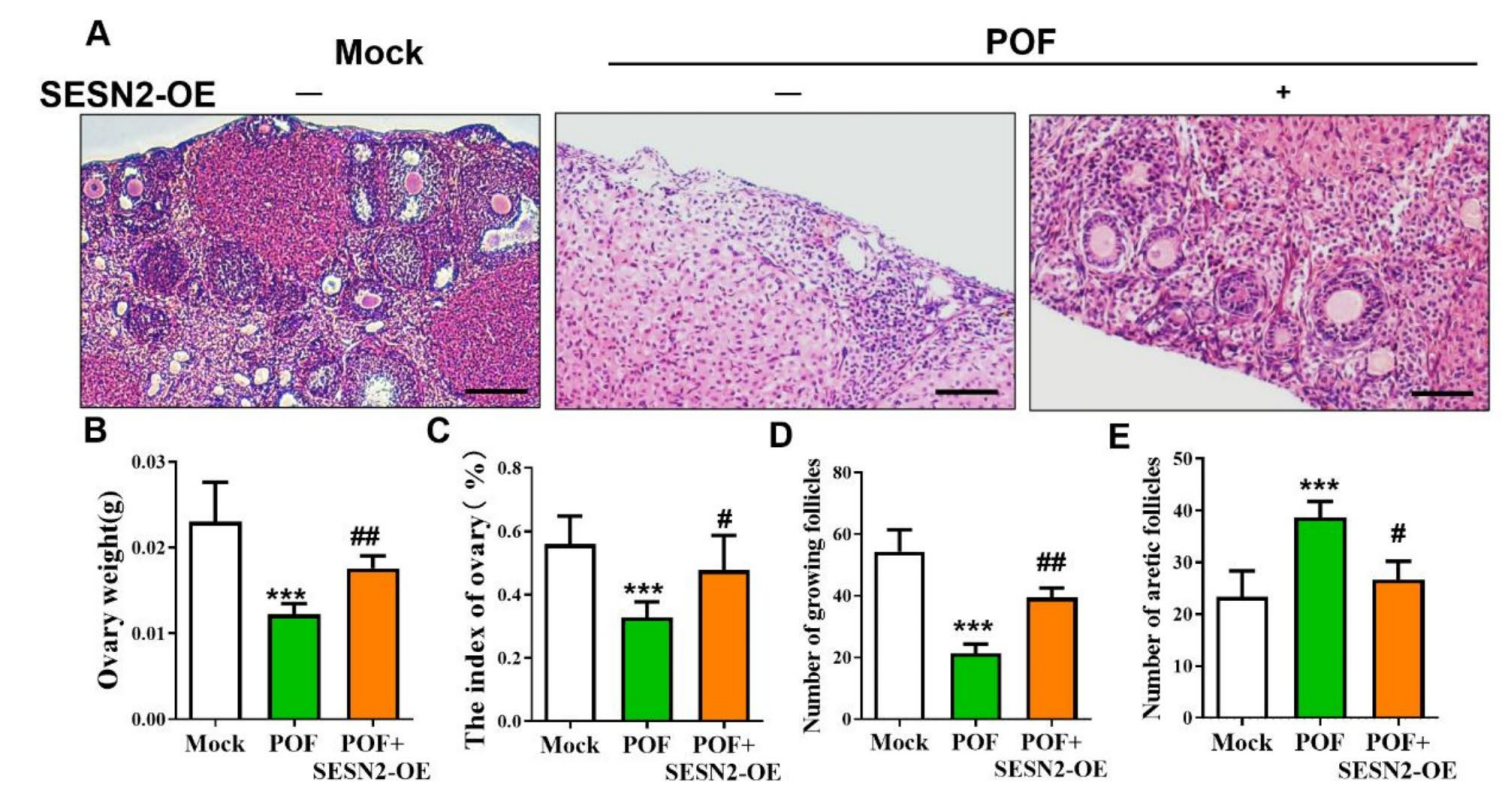
Effect of SESTRIN2 overexpression (SESN2-OE) on the ovarian reserve. (**A**) Representative H&E-stained images of murine ovaries; magnification; scale bar: 50 μm. (**B**, **C**) Ovary weights and ovarian indices of the mice in the Mock, POF, and POF + SESN2-OE groups (*n* = 5). (**D**, **E**) Follicle counting results according to ovary serial sections (*n* ≥ 4). All experiments were repeated at least three times, and the results of representative experiments are shown. **p < 0.01, ***p < 0.001, compared with the Mock group; ## *p* < 0.01, compared with the POF group; one-way ANOVA.

As shown in Fig. 5A, the results of HE staining revealed that the ovary structure of the mice in the POF group was severely damaged and that the number of follicles was significantly reduced, whereas the ovary structure of the mice in the SESN2-OE group was restored, and the number of follicles was significantly restored. Compared with those in the NC group, the changes in ovarian weight and indices in the POF group were significantly decreased, while those indicators in the SESN2-OE group were significantly increased (Fig. 5B, C; *P* < 0.05). Compared with the NC group, the POF group had fewer follicles at each developmental stage (primordial, prehierarchical, and total follicles) (*P* < 0.05) and had higher atretic follicle numbers (Fig. 5D, E; *P* < 0.05). Compared with those from the POF group, the layers from the SESN2-OE group had higher numbers of primordial, prehierarchical, and total follicles (*P* < 0.05), whereas SESN2-OE supplementation mitigated atretic follicle numbers and ovary apoptosis rates.

### Overexpression of SESTRIN2 inhibited apoptosis and promoted germ cell proliferation

**Fig. 6:**
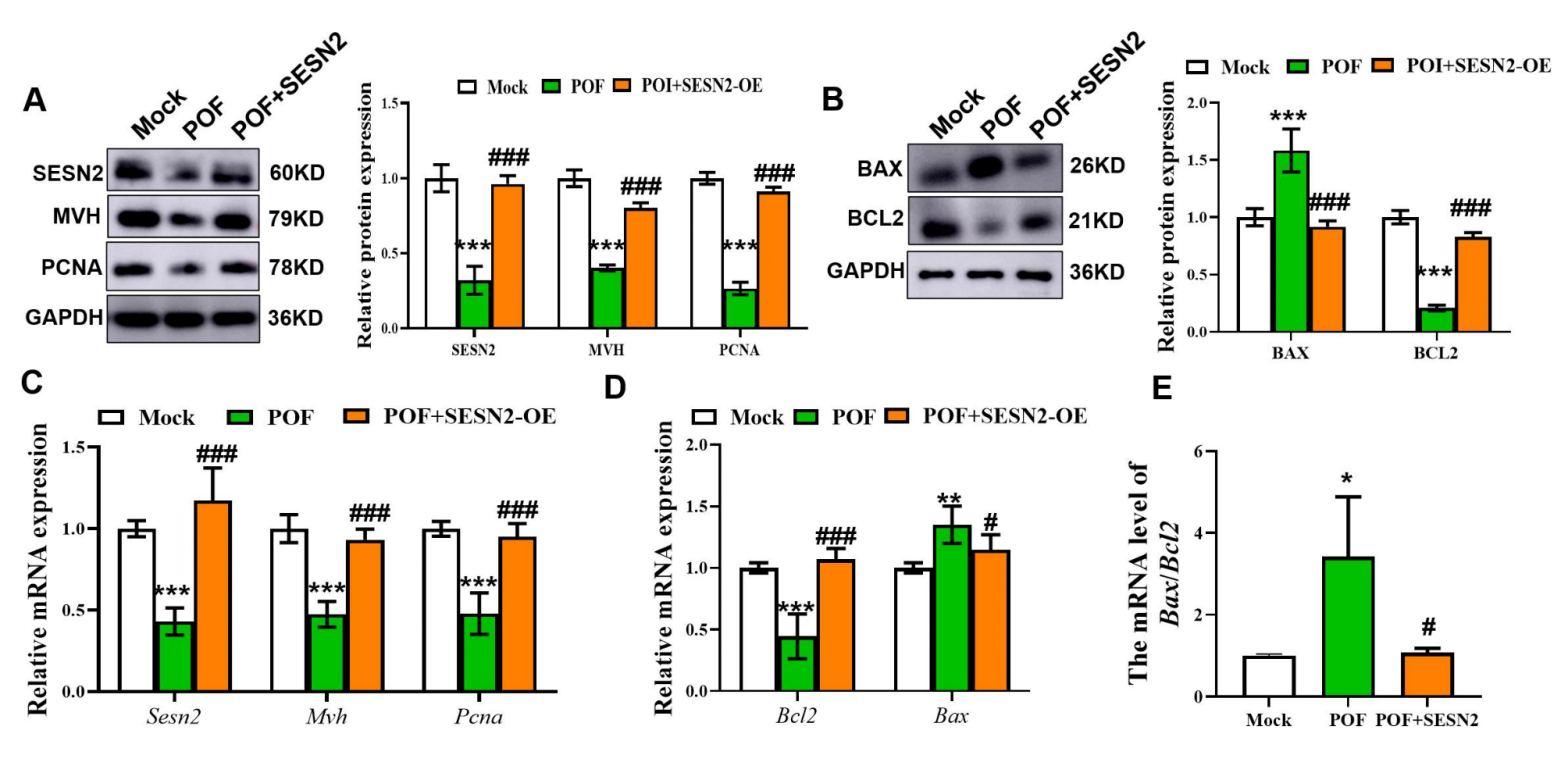
Effects of SESN2-OE on different treatment ovaries. (**A**) Expression levels of SESTRIN2-, MVH- and PCNA-related proteins in the Mock, POF, and POF + SESN2-OE groups (*n* = 5). (**B**) Expression levels of BAX- and BCL2-related proteins in the Mock, POF, and POF + SESN2-OE groups (*n* = 5). (**C**,** D**) Expression levels of *Sestrin2*, *Mvh*, *Pcna*,* Bax*, and *Bcl2* mRNAs in the Mock, POF, and POF + SESN2-OE groups. (**E**). The mRNA ratio of *Bax*/*Bcl2*. All experiments were repeated at least three times, and the results of representative experiments are shown. ** *p* < 0.01, *** *p* < 0.001, compared with the Mock group; # *p* < 0.05, ### *p* < 0.001, compared with the POF group; one-way ANOVA.

Compared with those in the Mock group, the protein and mRNA expression levels of MVH and PCNA in the POF group were significantly decreased, while the protein and mRNA expression levels of MVH and PCNA in the POF + SESN2-OE group were significantly increased (Fig. 6A, C; *p* < 0.001). As shown in Fig. 6. B, D, E, the protein and mRNA expression levels of BCL2 in the POF group were significantly lower than those in the Mock group, whereas the protein and mRNA expression levels of BAX were significantly increased, whereas the protein and mRNA expression levels of BCL2 in the POF + SESN2-OE group were significantly increased, whereas the protein and mRNA expression levels of BAX were significantly decreased in the POF group (Fig. 6B, D, E; *p* < 0.05). These results indicated that the overexpression of SESTRIN2 promoted germ cell proliferation and inhibited cell apoptosis.

**Fig. 7:**
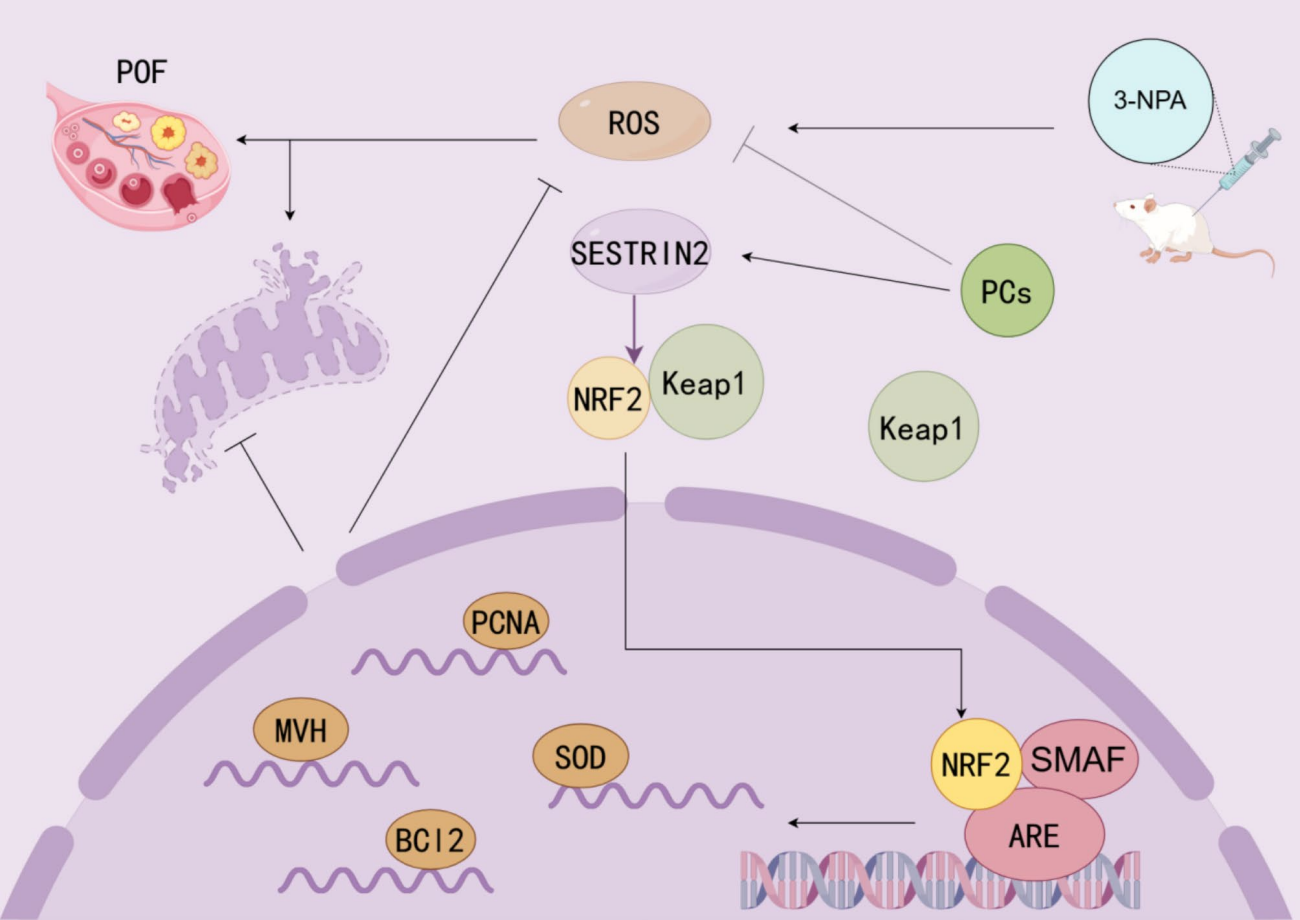
Proanthocyanidins regulate ovarian oxidative stress in mice with POF through the SESTRIN2-NRF2 pathway. 3-NPA induces excessive accumulation of ROS, which impairs mitochondrial function and eventually leads to POF. On the one hand, PCs directly remove ROS; on the other hand, they activate SESTRIN2 and then promote NRF2 and Keap1 separation and entry into the nucleus. In the nucleus, NRF2 promotes the expression of a variety of cytoprotective genes, which in turn cleans excessive ROS, protects mitochondrial function, and protects ovarian function.

## Discussion

Ovary function plays a central role in fertility, and ovarian function depends on the maintenance and normal development of ovarian follicles^49,50^. Accelerated metabolism occurs in rapidly proliferating granulosa cells (GCs) within developing follicles, leading to increased ROS production^50^. Normal levels of ROS are essential for the proper development of ovarian tissue, but excess ROS can trigger oxidative stress^51^. Accumulating evidence demonstrates that oxidative stress-induced granulosa cell (GCs) apoptosis is a common cause of follicular atresia^7,9^. Therefore, it is possible to treat follicular atresia by antagonizing oxidative stress with antioxidants. As powerful and easy-to-obtain antioxidants, proanthocyanidins have been used in a number of studies to treat diseases caused by antioxidative stress, but the mechanism is not clear. Therefore, this study aimed to explore the protective effect and mechanism of PCs in oxidative stress-induced premature ovarian failure and to evaluate the role of the SESTRIN2-NRF2 signaling pathway in alleviating and treating POF.

In this study, the mouse model of oxidative stress-induced premature ovarian failure was established via the intraperitoneal injection of 3-NPA. Compared with those in the control group, the mice in the 3-NPA group presented obvious ovarian atrophy, structural disorders, a significant reduction in the ovarian index and number of growing follicles, and a significant increase in the number of atretic follicles. In addition, the ROS and BAX levels in the ovaries of the 3-NPA group were significantly increased, whereas the MVH, PCNA, SOD and BCL2 levels were significantly decreased, indicating that the oxidative stress POF mice were successfully constructed. We then treated the mice with premature ovarian failure via the gavage of PCs. The results revealed that the ovarian index of the treatment group was significantly increased, the ovarian structure was significantly restored, the number of growth follicles at all levels was significantly increased, and the number of atretic follicles was significantly reduced. In addition, the ROS and BCL2 levels in the ovaries of the treatment group were significantly decreased, and the SOD, PCNA, MVH, and BAX levels were significantly increased, indicating that the oxidative stress and apoptosis of cells in the ovaries were effectively inhibited and that ovarian function was significantly restored, suggesting the potential of PCs as drugs for the treatment of premature ovarian failure caused by oxidative stress. Studies on the role of PCs in intestinal inflammation^52^, neuronal injury^53^, chronic respiratory diseases^54^ and other diseases have shown the effectiveness and safety of PCs.

Zhou et al. reported that PCs can remove free radicals and increase the expression of antioxidant enzymes by activating the Keap1-Nrf2/ARE signaling pathway^53^. NRF2 is separated from keap1 and enters the nucleus, where it binds to AREs and promotes the expression of downstream antioxidant genes, including SOD and GSH^39^. SOD is an enzyme that catalyzes the reduction of O2- to hydrogen peroxide. Glutathione catalyzes the reduction of hydrogen peroxide and other peroxides^55^. Ala et al. reported that SESTRIN2 also plays an important role in antioxidative stress, and one of its mechanism pathways is the activation of NRF2^56^. Interestingly, we found that SESTRIN2, NRF2, and SOD levels were significantly increased in the treatment group, so we hypothesized that PCs can counteract oxidative stress-induced apoptosis of ovarian cells by activating the SESTRIN2-NRF2 pathway. We next constructed a SESTRIN2 lentivirus overexpression vector and observed the ameliorative effect of this pathway on the POF model. After the transfection rate was verified by immunofluorescence and RT‒PCR, long-term stable expression of genes was guaranteed. The results revealed that the levels of E2 and FSH and the number of growing follicles in the SESTRIN2-OE group were significantly increased compared to POF group, which indicated that the ovarian function of the mice recovered significantly. The decreased levels of the oxidative stress products 4-HNE and 8-OHdG indicated that the damage to cells caused by oxidative stress was weakened.

In addition, with increasing SESTRIN2 activity, the estrous cycle, ovary weight and ovarian index of mice also change significantly, indicating that SESTRIN2 may affect the development, maturation and ovulation of mouse follicles by stimulating the secretion of related hormones in the endocrine system, such as the pituitary‒ovarian axis. In addition, the qPCR and WB results revealed that lentiviral overexpression of SESTRIN2 promoted germ cell proliferation. Therefore, our experiments verified the important role of SESTRIN2-KEAP1-NRF2 signaling pathway activation in the protection of ovarian function by PCs against oxidative stress, which also provides a potential pathway mechanism for the development of related drugs in the future.

However, 3-NPA (12.5 mg/kg/d) was intraperitoneally injected for 14 consecutive days to generate oxidative stress-induced premature ovarian failure in the mice in this study. Although many studies have used 3-NPA to generate oxidative stress-induced POF mice^41,57–59^, there are great differences in the dose of 3-NPA, the time of modeling, and the method of modeling. The doses of 3-NPA ranged from 6.25 to 40 mg/kg/day, and the duration of modeling ranged from 7 to 21 days. The methods of modeling were all intraperitoneal injection, but in some studies, the injections were performed twice a day. Therefore, the methods used to establish a 3-NPA-induced oxidative POF mouse model need to be unified. In addition, in this study, mouse fertility was used as an indicator to calculate the sample size in the preliminary experiment, but many observation indicators were used in this study, and whether the final sample size could meet the requirements of other indicators needs to be further calculated.

In summary, the SESTRIN2-KEAP1-NRF2 signaling pathway maintains ovarian proliferative activity and normal follicle development and plays a role in improving ovarian aging. PCs increase the activity of the SESTRIN2-KEAP1-NRF2 pathway, thereby protecting ovaries from oxidative stress (Fig. 7). Although the study of Zhang et al.^60^ verified the protective effect of proanthocyanidins on ovaries, no mechanism pathway was involved; however, this study revealed that the sestrin2-nrf2 signaling pathway, a key pathway of proanthocyanidins against oxidative stress, provides a theoretical basis for the future development of drugs for premature ovarian failure induced by oxidative stress.

## Electronic supplementary material

Below is the link to the electronic supplementary material.

Supplementary Material 1

## Data Availability

All raw data and materials are available from the corresponding author on reasonable request. All methods that were mentioned in this manuscript were performed in accordance with the relevant guidelines and regulations.
