# Supplementary material for: Proanthocyanidins protects 3-NPA-induced ovarian function decline by activating SESTRIN2-NRF2-mediated oxidative stress in mice
Source: Sci Rep. 2024 Oct 27;14:25643. doi: 10.1038/s41598-024-76743-w (PMC11514188; doi:10.1038/s41598-024-76743-w)
Supplement: Supplementary file 1 — Supplementary Material 1 [file 41598_2024_76743_MOESM1_ESM.pptx]

## Slide 1
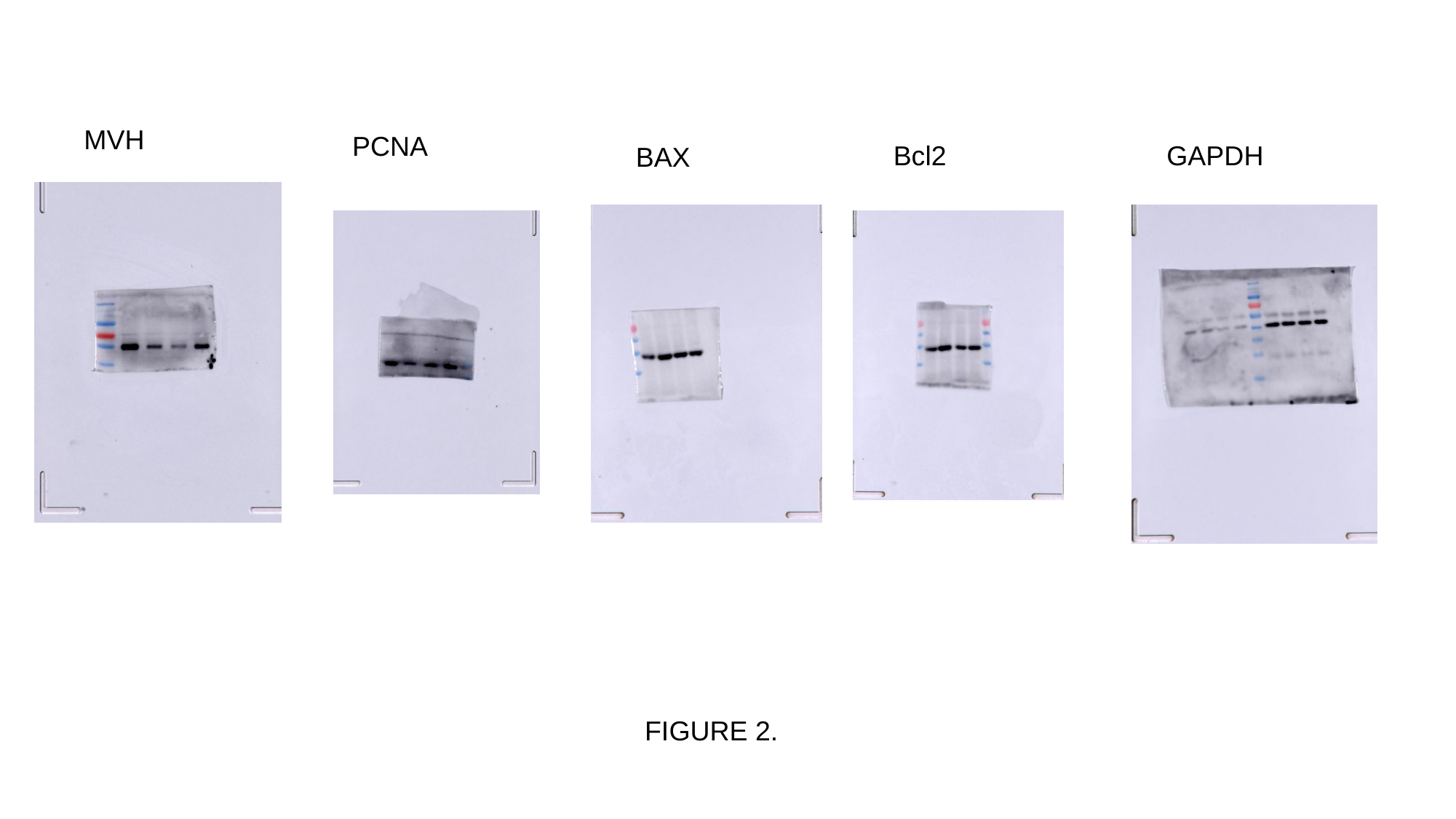

MVH
PCNA
Bcl2
GAPDH
BAX
FIGURE 2.

## Slide 2
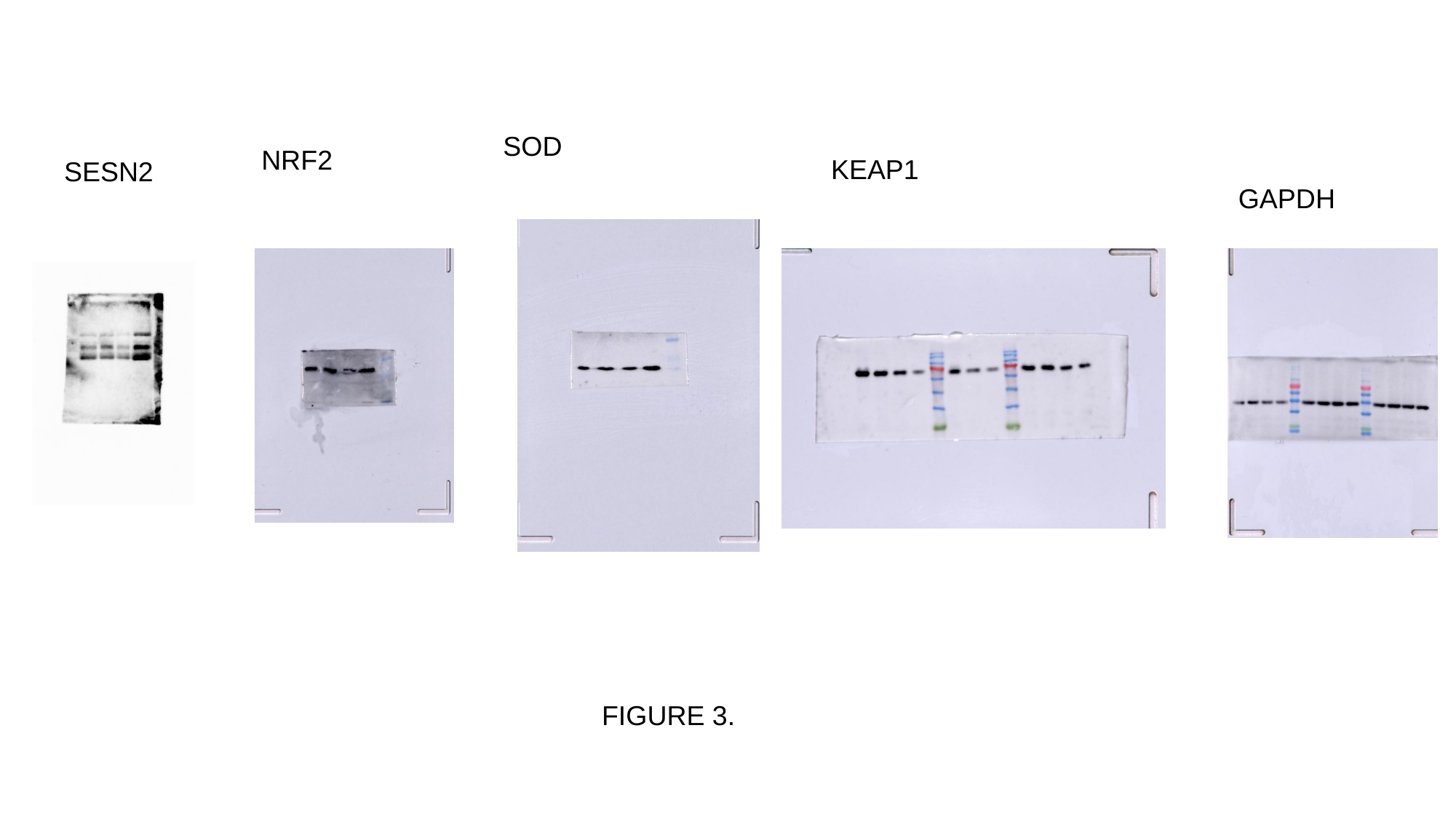

SOD
NRF2
KEAP1
SESN2
GAPDH
FIGURE 3.

## Slide 3
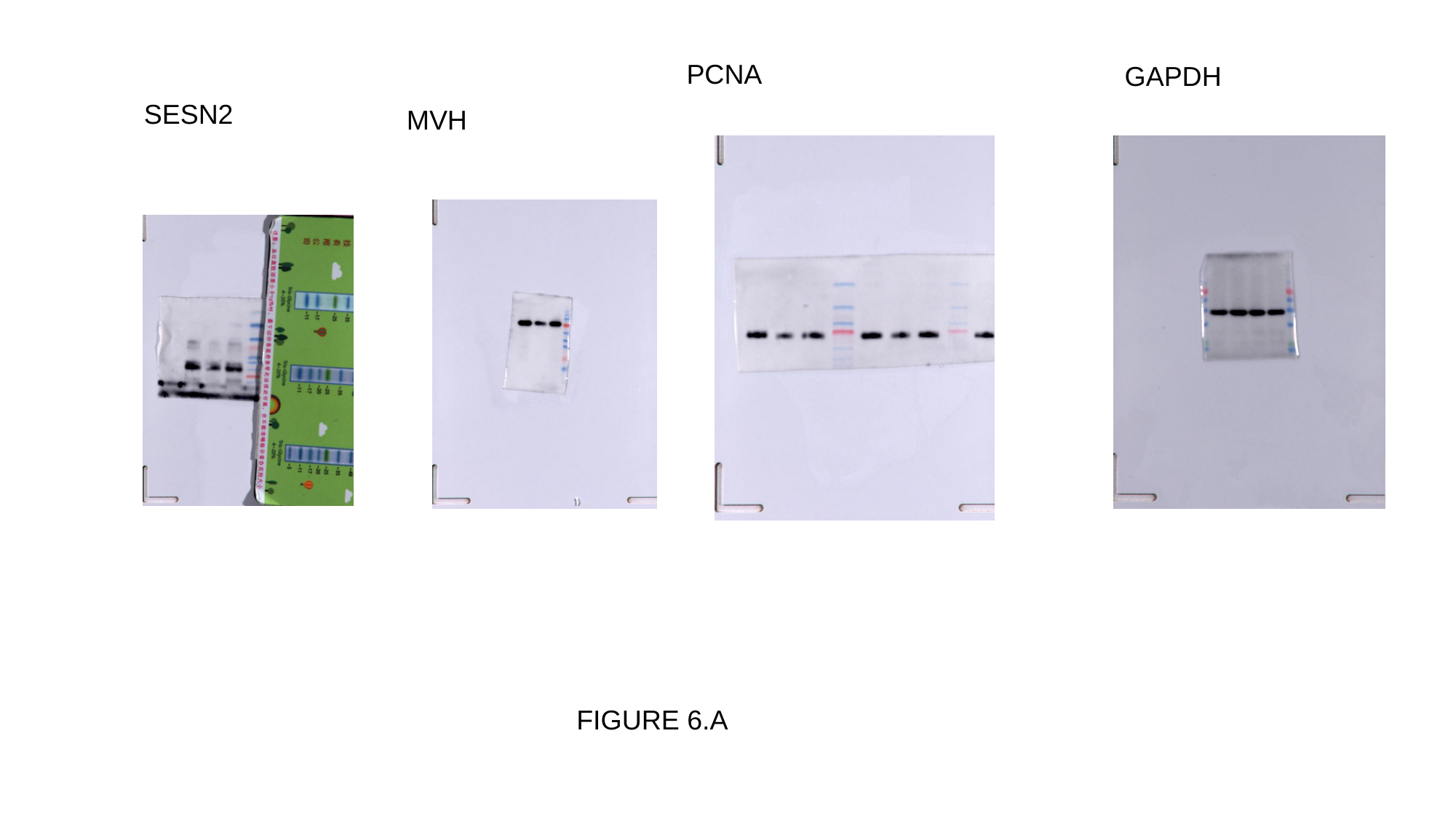

PCNA
GAPDH
SESN2
MVH
FIGURE 6.A

## Slide 4
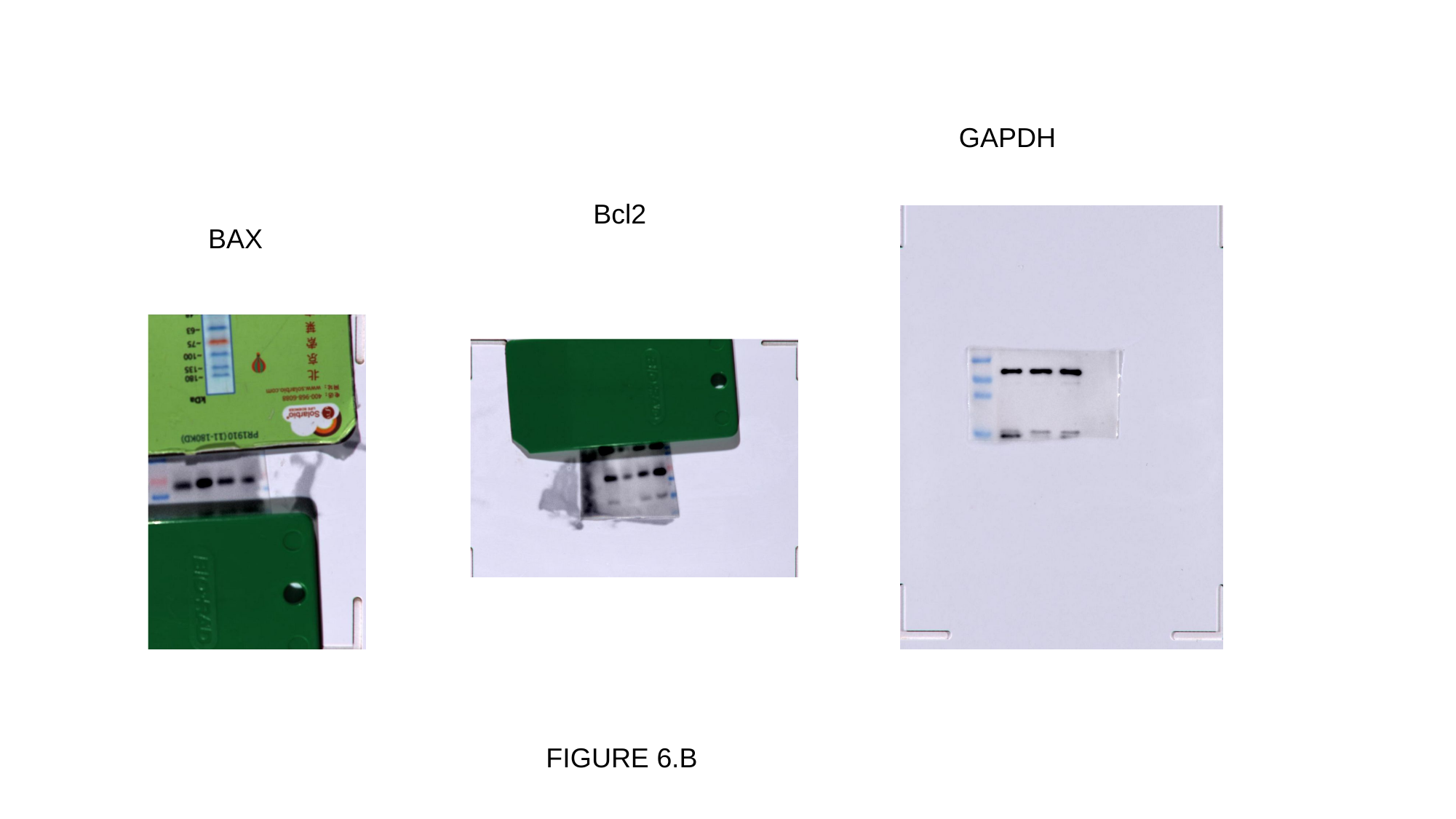

GAPDH
Bcl2
BAX
FIGURE 6.B
